# Real‐world data on EGFR/ALK gene status and first‐line targeted therapy rate in newly diagnosed advanced non‐small cell lung cancer patients in Northern China: A prospective observational study

**DOI:** 10.1111/1759-7714.13090

**Published:** 2019-05-29

**Authors:** Hongge Liang, Xia Song, Yuhui Zhang, Shucai Zhang, Fang Li, Jian Fang, Junling Li, Li Liang, Ligong Nie, Kewei Ma, Liangming Zhang, Xiaohong Wang, Junjun Xu, Yanxia Wei, Jinghui Wang, Qi Song, Guangming Tian, Yuxin Mu, Yangchun Gu, Lei Yang, Ping Sun, Wei Zhong, Jing Zhao, Yan Xu, Minjiang Chen, Mengzhao Wang

**Affiliations:** ^1^ Respiratory Medicine Peking Union Medical College Hospital Beijing China; ^2^ Respiratory Medicine Shanxi Provincial Cancer Hospital Taiyuan China; ^3^ Respiratory Medicine Beijing Chaoyang Hospital Beijing China; ^4^ Medical Oncology Beijing Chest Hospital, Capital Medical Hospital Beijing China; ^5^ Medical Oncology Military General Hospital of Beijing Beijing China; ^6^ Medical Oncology Beijing Cancer Hospital Beijing China; ^7^ Medical Oncology Chinese Academy of Medical Sciences Cancer Institute and Hospital Beijing China; ^8^ Medical Oncology Peking University Third Hospital Beijing China; ^9^ Respiratory Medicine Peking University First Hospital Beijing China; ^10^ Medical Oncology Jilin University First Hospital Changchun China; ^11^ Medical Oncology Qindao University Medical College Affiliated Yantai Yuhuangding Hospital Yantai China; ^12^ Medical Oncology Baotou Cancer Hospital Baotou China

**Keywords:** *ALK* rearrangement, *EGFR* mutation, evaluation status, non‐small cell lung cancer

## Abstract

**Background:**

Tyrosine kinase inhibitors (TKIs) can significantly prolong overall survival for patients with advanced non‐small cell lung cancer (NSCLC) harboring epidermal growth factor receptor (*EGFR*)‐mutation or anaplastic lymphoma kinase (*ALK*)‐rearrangement. However, the real‐world evaluation status of ALK/EGFR in China remains unclear.

**Methods:**

We conducted a prospective study including 1134 patients with cytologically or histologically confirmed advanced NSCLC (stage IIIb–IV) at 12 Chinese hospitals.

**Results:**

The most common evaluation methods were amplification‐refractory mutation system for *EGFR* status and immunohistochemistry targeting D5F3 for *ALK* status. Among patients with non‐squamous, the *EGFR* mutation rate was 44.1% and the *ALK* rearrangement rate was 10.0%. Among patients with squamous cell carcinoma, the *EGFR* mutation rate was 8.3% and the *ALK* rearrangement rate was 3.7%. Among all patients, gender (HR = 1.7, 95%CI = 1.2–2.4, *P* = 0.006), smoking history (HR = 1.8, 95%CI = 1.3–2.7, *P* = 0.001), histology (HR = 5.0, 95%CI = 2.4–10.1, *P* < 0.001), and brain metastases (HR = 1.5, 95%CI = 1.1–2.2, *P* = 0.017) were independent predictors of *EGFR* mutation, while age (HR = 2.6, 95%CI = 1.7–4.1, *P* < 0.001) was an independent predictor of *ALK* rearrangement. The median time from tumor diagnosis to *EGFR* or *ALK* status confirmation was 7 and 5 days, respectively. Targeted therapy rate was 73.8% in *EGFR*‐positive patients and 51.4% in *ALK*‐positive patients. There was a negative correlation between the first‐line targeted therapy rate and the *EGFR* mutation detection period (r = −0.152, *P* = 0.02), while no significant correlation among patients with *ALK* rearrangement (r = −0.179, *P* = 0.076).

**Conclusion:**

Squamous NSCLC patients should also be routinely tested to determine their *EGFR*/*ALK* statuses. The first‐line targeted therapy rate remains low in Chinese patients with NSCLC.

## Introduction

Lung cancer has become one of the most common cancers worldwide, with high morbidity and mortality rates as most patients are not eligible for radical surgery at the time of diagnosis. Furthermore, traditional radiotherapy and chemotherapy have limited effects in cases of non‐small cell lung cancer (NSCLC). Recent research regarding targeted therapy such as epidermal growth factor receptor‐tyrosine kinase inhibitors (*EGFR*‐TKIs) and anaplastic lymphoma kinase inhibitors (*ALK*‐TKIs) has revealed significant improvements in overall survival rates of NSCLC patients harboring *EGFR* mutation or *ALK* rearrangement. As a result, targeted therapy has been approved as a first‐line treatment for these patients.^1^, ^2^, ^3^, ^4^, ^5^, ^6^, ^7^, ^8^, ^9^, ^10^, ^11^, ^12^

Several studies have been performed to determine the current status of *EGFR‐*mutation/*ALK*‐rearrangement in Asia, including China. For example, Pan *et al.* 13 analyzed 176 NSCLC patients treated at the First Affiliated Hospital of Wenzhou Medical College, and observed that the total mutation rate of the *EGFR* gene in exons 19, 20, and 21 was 48.3% (85/176). They further identified several factors, including female gender, adenocarcinoma, distant metastasis, and the chemotherapy, that may increase the probability of *EGFR* gene mutations. Shi *et al.* 14 analyzed 747 patients with advanced NSCLC among a subset of patients from mainland China with an adenocarcinoma history as part of the PIONEER study, and found that the overall *EGFR* mutation rate was 50.2% among the 741 patients that were successfully genotyped, while the activating *EGFR* mutation rate was 48.0% (with 1.3% of patients showing combined activating and resistance mutations). Smoking history and regional lymph nodes involvement were identified as independent predictors of *EGFR* mutation in multivariate analysis. Zhou *et al.* 15 analyzed *EGFR* mutations of 261 patients with pathologically confirmed NSCLC from West China Hospital, and observed that the *EGFR* mutation rate was 48.7%, with smoking status and pathological types as independent predictors. Fu *et al.* 16 recruited 487 lung cancer patients who underwent testing for *ALK* rearrangement at Sun Yat‐sen University Cancer Center, and found that the *ALK* rearrangement rate was 9.0% (44/487), and that *ALK*‐rearranged NSCLC tended to occur in younger individuals who were either non‐smokers or light smokers with adenocarcinoma.

However, the current evaluation methods and periods of *EGFR* mutation and*/*or *ALK* rearrangement, as well as the first‐line targeted therapy rate in patients with NSCLC harboring *EGFR* mutations or *ALK* rearrangement in China remain unclear. Moreover, previous studies have been limited by their retrospective design. Therefore, we conducted a prospective multicenter study with the goal of determining the detection methods and detection periods of *EGFR* mutation and *ALK* rearrangement, the *EGFR* mutation rate, *ALK* rearrangement rate, and first‐line targeted therapy rate in patients with NSCLC harboring *EGFR* mutations or *ALK* rearrangement in northern China.

## Methods

### Study design and patients

We conducted a prospective, epidemiological, multicenter, pan‐label, and non‐comparative study of *EGFR* mutation and *ALK* rearrangement evaluation status, and first‐line targeted therapy rate of patients with newly diagnosed advanced (stage IIIb–IV) NSCLC. This study only involved an observational protocol, and did not affect the patients’ diagnosis and treatment. The study protocol was approved by the participating institutions’ ethics committees. Patients who were eligible for enrollment provided written informed consent for participation in the study.

Patients with locally advanced or metastatic NSCLC (stage IIIb–IV) were enrolled at 12 hospitals in northern China between March 2015 and April 2017. The inclusion criteria were: (i) age ≥ 18 years, (ii) new diagnosis of NSCLC confirmed using histology or cytology, (iii) locally advanced or metastatic NSCLC (stage IIIb–IV or recurrent cases that were not eligible for surgery or radical chemoradiotherapy), (iv) simultaneous results for *EGFR* mutation and *ALK* rearrangement testing, and (v) no previous systemic treatment (except adjuvant chemotherapy). The exclusion criteria were: (i) previous non‐adjuvant systemic treatment, (iii) only sputum pathology specimens available, (iv) genetic results from sputum or blood samples, and (v) gene testing methods that did not fulfill the inclusion criteria.

### Data collection

Demographic and clinical characteristics of patients were collected, including age at diagnosis, gender, smoking status, date of first pathological diagnosis, method of pathological diagnosis, date that the first *EGFR* mutation and *ALK* rearrangement was confirmed, *EGFR*‐mutation/*ALK*‐rearrangement detection period (time from tumor diagnosis to *EGFR/ALK* status confirmation), detection method of *EGFR* mutation and *ALK* rearrangement, distant metastases, and first‐line treatment.

### *EGFR*‐mutation and *ALK*‐rearrangement analysis

Tumor samples were obtained from primary or metastatic lesions, handled and stored following the respective laboratories’ quality control requirements. The *EGFR*‐mutation was analyzed by amplification refractory mutation system (ARMS) or next‐generation sequencing (NGS), whereas the *ALK*‐rearrangement was analyzed by fluorescence *in situ* hybridization (FISH), NGS, or Ventana immunohistochemistry (IHC) targeting D5F3.

### Statistical analyses

All statistical analyses were performed using IBM SPSS software (version 21.0; IBM Corp., Armonk, NY, USA). Continuous variables are expressed as the means ± standard deviation. Associations between mutations and demographic and clinical characteristics were analyzed by Fisher's exact tests. Characteristics significantly (*P* < 0.05) associated with mutations were then included in a multivariate logistic model. The hazard ratio (HR) and 95%CI were calculated for all variables in the regression model. The correlation between first‐line targeted therapy and the *EGFR/ALK* detection period were analyzed by Spearman's correlation. All tests were two‐sided, and statistical significance was set at *P* < 0.05. It is noteworthy that some patients underwent radical treatment and subsequently underwent *EGFR/ELK* gene testing several years after the diagnosis, which would not accurately reflect the *EGFR/ALK* detection period. Thus, the data from these cases were omitted from the related analyses of *EGFR/ALK* detection period.

## Results

### Patient characteristics

Between March 2015 and April 2017, a total of 1134 patients with cytologically or histologically confirmed advanced NSCLC (stage IIIb–IV) were enrolled in the study at 12 Chinese hospitals. Among these patients, the most common pathological type was adenocarcinoma (973 cases), followed by squamous cell carcinoma (109 cases), unclassified carcinoma (36 cases), adenosquamous carcinoma (11 cases), sarcomatoid carcinoma (3 cases), and large cell carcinoma (2 cases). The specimens were evaluated using histology (976 cases) and cytology (158 cases). The cases involved primary lesions (757 cases) or metastatic lesions (377 cases), including 172 cases of distal/local lymph node metastases, 126 cases of pleural effusion, 25 cases of pleural metastasis, 21 cases of bone metastasis, 10 cases of liver metastasis, eight cases of brain metastasis, seven cases of subcutaneous nodule metastasis, and eight cases of other metastatic sites. The most common biopsy methods were bronchoscopy (395 cases) and computed tomography‐guided lung puncture (365 cases). The other cases involved ultrasound‐guided puncture (191 cases), bone biopsy (16 cases), surgical biopsy (133 cases), radical resection (32 cases), and cerebrospinal fluid collection (2 cases). The most common biopsy site was the lung (66.8%), the most common biopsy method was bronchoscopy (34.6%), and the most common metastatic site was the lung (22.6%). The cases of squamous NSCLC patients frequently involved male patients (92/109) who were >60 years old (70/109), and patients with a smoking history (86/109). The cases of non‐squamous NSCLC patients involved male patients (550/1025) with a smoking history (430/1025), and patients who were >60 years old (516/1025). Table 1 summarizes the clinicopathological features of 1134 NSCLC patients, 1025 non‐squamous NSCLC patients, and 109 squamous NSCLC patients.

**Table 1:** Clinical and pathological features of 1134 NSCLC patients

	All patients	Non‐squamous	Squamous
Clinicopathology	No. (%)	No. (%)	No. (%)
Age			
Medium (Range)	60 (21–88)	60 (21–88)	63 (42–87)
≤60 years old	548 (48.4%)	509 (49.7%)	39 (35.8%)
>60 years old	586 (51.6%)	516 (50.3%)	70 (64.2%)
Gender			
Male	642 (56.6%)	550 (53.7%)	92 (84.4%)
Female	492 (43.4%)	475 (46.3%)	17 (15.6%)
Smoking history			
No	588 (51.9%)	565 (55.1%)	23 (21.1%)
Yes	515 (45.4%)	430 (42.0%)	85 (78.0%)
Unknown	31 (2.7%)	30 (2.9%)	1 (0.9%)
Stage			
IIIb	165 (14.6%)	120 (11.7%)	45 (41.3%)
IV	969 (85.4%)	905 (88.3%)	64 (58.7%)
Pathology			
Non‐squamous	1025 (90.4%)	1025 (100.0%)	0 (0.0%)
Squamous	109 (9.6%)	0 (0.0%)	109 (100.0%)
Diagnostic methods			
Histology	976 (86.1%)	867 (84.6%)	109 (100.0%)
cytology	158 (13.9%)	158 (15.4%)	0 (0.0%)
EGFR mutation			
Mutant type	461 (40.7%)	452 (44.1%)	9 (8.3%)
Wild type	673 (59.3%)	573 (55.9%)	100 (91.7%)
ALK rearrangement			
Mutant type	107 (9.4%)	103 (10.0%)	4 (3.7%)
Wild type	1027 (90.6%)	922 (90.0%)	105 (96.3%)

EGFR, epidermal growth factor receptor; ALK, anaplastic lymphoma kinase.

### *EGFR/ALK* evaluation status

Among all patients, the most commonly used methods of detection for *EGFR* mutation and *ALK* rearrangement were ARMS (1029/1134, 90.7%) and IHC targeting D5F3 (692/1134, 61.0%), respectively. Among patients with non‐squamous NSCLC, the most commonly used methods of detection for *EGFR* mutation and *ALK* rearrangement were ARMS (933/1025, 91.0%) and IHC targeting D5F3 (637/1025, 62.1%), respectively. Six cases were evaluated for *ALK* rearrangement using both NGS and IHC (2 cases) or both ARMS and IHC (4 cases), which revealed consistent findings. Twenty‐two cases were evaluated for *ALK* rearrangement using both FISH and IHC, which revealed consistent findings in 21 cases and inconsistent findings in one case (positive IHC results and negative FISH results). Among patients with squamous NSCLC, the most commonly used methods of detection for *EGFR* mutation and *ALK* rearrangement were ARMS (96/109 88.1%) and IHC targeting D5F3 (55/109, 50.1%), respectively. One cases were evaluated for *ALK* rearrangement using both ARMS and IHC, which revealed consistent findings. Two cases were evaluated for *ALK* rearrangement using both FISH and IHC, which revealed inconsistent findings (positive IHC results and negative FISH results).

The median time from the biopsy to tumor diagnosis was three days (range: 0–54 days). Sixty patients underwent genetic testing several years after undergoing radical therapy, which would not accurately reflect the *EGFR/ALK* detection period; thus, these cases were omitted from the following analyses. Among the remaining cases, the median time from tumor diagnosis to *EGFR* status confirmation was seven days (range: 0–84 days), with median times of seven days (range: 0–84 days) for ARMS and nine days (range: 0–42 days) for NGS. The median time from tumor diagnosis to *ALK* status confirmation was five days (range: 0–81 days), with median times of seven days (range: 0–81 days) for NGS, seven days (range: 0–55 days) for FISH, and four days (range: 0–42 days) for IHC.

### *EGFR/ALK* evaluation results

Among all patients, the *EGFR* mutation rate was 40.7% (461/1134), with the major mutations being 19del and 21L858R. The sensitive mutation rate was 38.8% (440/1134) and the primary drug‐resistant mutation rate was 1.8% (21/1134). Nineteen patients (1.7%) had double *EGFR* mutations, with the most common being L858R and T790M (8/19) (Table 2). The *EML4‐ALK* rearrangement rate was 9.4% (107/1134), which included 100 adenocarcinomas, four squamous cell carcinomas, two adenosquamous carcinomas, and one NSCLC. Among patients with non‐squamous, the *EGFR* mutation rate was 44.1% (452/1025) and the *ALK* rearrangement rate was 10.0% (103/1025). Among patients with squamous cell carcinoma, the EGFR mutation rate was 8.3% (9/109) and the ALK rearrangement rate was 3.7% (4/109).

**Table 2:** Mutation patterns of 19 cases with EGFR double mutations

Pts.	19del	L858R	L861Q	G719X	S768I	T790M	20ins	G	Age	SH	P	TNM stage
1	−	−	−	+	+	−	−	M	76	N	AS	T2N3M0
2	−	−	−	+	+	−	−	F	54	N	A	T1N0M1
3	−	−	−	+	+	−	−	M	60	Y	A	T4N2M1
4	−	−	−	+	+	−	−	M	49	Y	A	T2N3M1
5	−	−	−	+	+	−	−	F	51	N	A	T2N3M1
6	+	−	−	+	−	−	−	F	68	Y	AS	T4N3M0
7	+	−	−	−	−	+	−	F	58	Y	A	TXNXM1
8	+	−	−	−	−	+	−	F	80	Y	A	T2N2M1
9	+	+	−	−	−	−	−	F	65	Y	A	T2N0M1
10		+	−	−	+	−	−	M	71	Y	A	T1N3M0
11		+	−	−	+	−	−	M	59	Y	A	T1N3M1
12		+	−	−	−	+	−	M	64	Y	A	T4N3M1
13		+	−	−	−	+	−	M	66	Y	S	T4N2M1
14		+	−	−	−	+	−	F	60	N	A	T1N1M1
15		+	−	−	−	+	−	F	76	N	A	T4N3M1
16		+	−	−	−	+	−	F	50	N	A	T3N2M1
17		+	−	−	−	+	−	M	46	Y	A	T2N2M1
18		+	−	−	−	+	−	F	76	N	A	T4N0M1
19		+	−	−	−	+	−	M	66	N	A	T2N0M1

A, adenocarcinoma; AS, adenosquamous carcinoma; F, female; G, gender; M, male; N, no, never smoker; P, pathology; S, squamous carcinoma; SH, smoking history; Y, yes, former or current smoker.

Among all patients, univariate analyses showed that the *EGFR* mutation rate was significantly higher in females (*P* < 0.001), without a smoking history (*P* < 0.001), non‐squamous (*P* < 0.001), stage IV tumor (*P* < 0.001), bone metastases (*P* = 0.014), brain metastases (*P* = 0.002), pleural effusion (*P* = 0.016) and pleural nodules (*P* = 0.014) (Table 3). Multivariate analysis further identified gender (HR = 1.7, 95%CI = 1.2–2.4, *P* = 0.006), smoking history (HR = 1.8, 95%CI = 1.3–2.7, *P* = 0.001), histology (HR = 5.0, 95%CI = 2.4–10.1, *P* < 0.001), and brain metastases (HR = 1.5, 95%CI = 1.1–2.2, *P* = 0.017) as independent predictors of *EGFR* mutation. Among patients with non‐squamous , univariate analyses showed that the *EGFR* mutation rate was significantly higher in females (*P* < 0.001), without a smoking history (*P* < 0.001), stage IV tumor (*P* = 0.002), brain metastases (*P* = 0.027), and pleural nodules metastases (*P* = 0.034) (Table 4). Multivariate analysis further identified female (HR = 1.6, 95%CI = 1.1–2.3, *P* = 0.013), without a smoking history (HR = 1.9, 95%CI = 1.3–2.7, *P* = 0.001), and brain metastases (HR = 1.5, 95%CI = 1.1–2.1, *P* = 0.021) had higher *EGFR* mutation. Among patients with squamous, univariate analyses showed that the *EGFR* mutation rate was significantly higher in females (*P* = 0.004), without a smoking history (*P* = 0.049), and pleural effusion (*P* = 0.032) (Table 5). Multivariate analysis further identified only gender (HR = 6.0, 95%CI = 1.1–32.6, *P* = 0.040) as independent predictor of *EGFR* mutation.

**Table 3:** Fisher exact probability method analysis of EGFR mutation and clinical characteristics in 1134 NSCLC patients

Characteristics	No. (%)	Wild type (%)	Mutant type (%)	*P*‐value
Age				
<60 years old	548 (48.3%)	317 (57.8%)	231 (42.2%)	0.333
≥60 years old	586 (51.7%)	356 (60.8%)	230 (39.2%)	
Gender				
Male	642 (56.6%)	450 (70.1%)	192 (29.9%)	<0.001
Female	492 (43.4%)	223 (45.3%)	269 (54.7%)	
Smoking history				
No	588 (53.3%)	282 (48.0%)	30 (52.0%)	<0.001
Yes	515 (46.7%)	380 (73.8%)	135 (26.2%)	
Unknown	31	11	20	
Pathology				
Non‐squamous	1025 (90.4%)	573 (55.9%)	452 (44.1%)	<0.001
Squamous	109 (9.6%)	100 (91.7%)	9 (8.3%)	
T stage				
1	153 (13.5%)	83 (54.2%)	70 (45.8%)	0.093
2	268 (23.6%)	154 (57.5%)	114 (42.5%)	
3	107 (9.4%)	72 (67.3%)	35 (32.7%)	
4	526 (46.4%)	323 (61.4%)	203 (38.6%)	
x	80 (7.1%)	41 (51.3)	39 (48.8)	
N stage				
0	161 (14.2%)	82 (50.9%)	79 (49.1%)	0.058
1	48 (4.2%)	26 (54.2%)	22 (45.8%)	
2	298 (26.3%)	174 (58.4%)	124 (41.6%)	
3	593 (52.3%)	373 (62.9%)	220 (37.1%)	
x	34 (3.0%)	18 (52.9%)	16 (47.1%)	
M stage				
0	166 (14.6%)	126 (75.9%)	40 (24.1%)	<0.001
1	968 (85.4%)	547 (56.5%)	421 (43.5%)	
Lung metastases				
No	724 (63.8%)	442 (61.0%)	282 (39.0%)	0.131
Yes	410 (36.2%)	231 (56.3%)	179 (43.7%)	
Bone metastases				
No	733 (64.6%)	455 (62.1%)	278 (37.9%)	0.014
Yes	401 (35.4%)	218 (54.4%)	183 (45.6%)	
Brain metastases				
No	948 (16.4%)	582 (61.4%)	366 (38.6%)	0.002
Yes	186 (83.6%)	91 (48.9%)	95 (51.1%)	
Adrenal metastases				
No	1046 (92.2%)	613 (58.6%)	433 (41.4%)	0.090
Yes	88 (7.8%)	60 (68.2%)	28 (31.8%)	
Liver metastases				
No	1040 (91.7%)	610 (58.7%)	430 (41.3%)	0.125
Yes	94 (8.3%)	63 (67.0%)	31 (33.0%)	
Pleural effusion				
No	813 (71.7%)	501 (61.6%)	312 (38.4%)	0.016
Yes	321 (28.3%)	172 (53.6%)	149 (46.4%)	
Pleural nodules				
No	1002 (88.4%)	608 (60.7%)	394 (39.3%)	0.014
Yes	132 (11.6%)	65 (49.2%)	67 (50.8%)	

EGFR, epidermal growth factor receptor.

**Table 4:** Fisher exact probability method analysis of EGFR mutation and clinical characteristics in 1025 non‐squamous NSCLC patients

characteristics	No.	Wild type	Mutant type	*P*‐value
Age (N,%)				
<60 years old	509 (49.7%)	285 (56.0%)	224 (44.0%)	1.000
≥60 years old	516 (50.8%)	288 (55.8%)	228 (44.2%)	
Gender (N,%)				
Male	550 (53.7%)	362 (65.8%)	188 (34.2%)	<0.001
Female	475 (46.3%)	211 (44.4%)	264 (55.6%)	
Smoking history (N,%)				
No	565 (55.1%)	264 (46.7%)	301 (53.3%)	<0.001
Yes	430 (42.0%)	299 (69.5%))	131 (30.5%)	
Unknown	30 (2.9%)	10 (33.3%)	20 (66.7%)	
T stage (N,%)				
1	148 (14.4%)	78 (52.7%)	70 (47.3%)	0.415
2	247 (24.1%)	136 (55.1%)	111 (44.9%)	
3	96 (9.4%)	61 (63.5%)	35 (36.5%)	
4	457 (44.6%)	259 (56.7%)	198 (43.3%)	
x	77 (7.5%)	39 (50.6%)	38 (49.4%)	
N stage (N,%)				
0	154 (15.0%)	75 (48.7%)	79 (51.3%)	0.107
1	42 (4.1%)	21 (50.0%)	21 (50.0%)	
2	263 (25.7%)	142 (54.0%)	121 (46.0%)	
3	533 (52.0%)	318 (59.7%)	215 (40.3%)	
x	33 (3.2%)	17 (51.5%)	16 (48.5%)	
M stage (N,%)				
0	121 (14.6%)	84 (69.4%)	37 (30.6%)	0.002
1	904 (85.4%)	489 (54.1%)	415 (45.9%)	
Lung metastases				
No	645 (62.9%)	370 (57.4%)	275 (42.6%)	0.241
Yes	380 (37.1%)	203 (53.4%)	177 (46.6%)	
Bone metastases				
No	650 (63.4%)	378 (58.2%)	272 (41.8%)	0.058
Yes	375 (36.6%)	195 (52.0%)	180 (48.0%)	
Brain metastases				
No	841 (82.0%)	484 (57.6%)	357 (42.4%)	0.027
Yes	184 (18.0%)	89 (48.4%)	95 (51.6%)	
Adrenal metastases				
No	942 (91.9%)	518 (55.0%)	424 (45.0%)	0.050
Yes	83 (8.1%)	55 (66.3%)	28 (33.7%)	
Liver metastases				
No	938 (91.5%)	516 (55.0%)	422 (45.0%)	0.071
Yes	87 (8.5%)	57 (65.5%)	30 (34.5%)	
Pleural effusion				
No	721 (70.3%)	414 (57.4%)	307 (42.6%)	0.148
Yes	304 (29.7%)	159 (52.3%)	145 (47.7%)	
Pleural nodules				
No	901 (87.9%)	515 (57.2%)	386 (42.8%)	0.034
Yes	124 (12.1%)	58 (46.8%)	66 (53.2%)	

EGFR, epidermal growth factor receptor.

**Table 5:** Fisher exact probability method analysis of EGFR mutation and clinical characteristics in 109 squamous NSCLC patients

Characteristics	No.	Wild type	Mutant type	*P*‐value
Age (N,%)				
<60 years old	39 (35.8%)	34 (87.2%)	5 (12.8%)	0.277
≥60 years old	70 (64.2%)	66 (94.3%)	4 (5.7%)	
Gender (N,%)				
Male	92 (84.4%)	88 (95.7%)	4 (4.3%)	0.004
Female	17 (15.6%)	12 (70.6%)	5 (29.4%)	
Smoking history (N,%)				
No	23 (21.1%)	18 (78.3%)	5 (21.7%)	0.049
Yes	85 (78.0%)	81 (95.3%))	4 (4.7%)	
Unknown	1 (0.9%)	1 (100.0%)	0 (0.0%)	
T stage (N,%)				
1	5 (4.6%)	5 (100.0%)	0 (0.0%)	0.287
2	21 (19.3%)	18 (85.7%)	3 (14.3%)	
3	11 (10.1%)	11 (100.0%)	0 (0.0%)	
4	69 (63.3%)	64 (92.8%)	5 (7.2%)	
x	3 (2.8%)	2 (66.7%)	1 (33.3%)	
N stage (N,%)				
0	7 (6.4%)	7 (100.0%)	0 (0.0%)	0.743
1	6 (5.5%)	5 (83.3%)	1 (16.7%)	
2	35 (32.1%)	32 (91.4%)	3 (8.6%)	
3	60 (55.0%)	55 (91.7%)	5 (8.3%)	
x	1 (0.9%)	1 (100.0%)	0 (0.0%)	
M stage (N,%)				
0	45 (41.3%)	42 (93.3%)	3 (6.7%)	0.734
1	64 (58.7%)	58 (90.6%)	6 (9.4%)	
Lung metastases				
No	79 (72.5%)	72 (91.1%)	7 (8.9%)	1.000
Yes	30 (27.5%)	28 (93.3%)	2 (6.7%)	
Bone metastases				
No	83 (76.1%)	77 (92.8%)	6 (7.2%)	0.443
Yes	26 (23.9%)	23 (88.5%)	3 (11.5%)	
Brain metastases				
No	107 (98.2%)	98 (91.6%)	9 (8.4%)	1.000
Yes	2 (1.8%)	2 (100.0%)	0 (0.0%)	
Adrenal metastases				
No	104 (95.4%)	95 (91.3%)	9 (8.7%)	1.000
Yes	5 (4.6%)	5 (100.0%)	0 (0.0%)	
Liver metastases				
No	102 (93.6%)	94 (92.2%)	8 (7.8%)	0.463
Yes	7 (6.4%)	6 (85.7%)	1 (14.3%)	
Pleural effusion				
No	92 (84.4%)	87 (94.6%)	5 (5.4%)	0.032
Yes	17 (15.6%)	13 (76.5%)	4 (23.5%)	
Pleural nodules				
No	101 (92.7%)	93 (92.1%)	8 (7.9%)	0.510
Yes	8 (7.3%)	7 (87.5%)	1 (12.5%)	

EGFR, epidermal growth factor receptor.

Among all patients, univariate analyses showed that the *ALK* rearrangement rate was significantly higher in patients who were ≤60 years old (*P* < 0.001), female (*P* = 0.002), without a smoking history (*P* < 0.001), and non‐squamous (*P* = 0.025) (Table 6). In multivariate analysis, only younger age (HR = 2.6, 95%CI = 1.7–4.1, *P* < 0.001) was an independent predictor of *ALK* rearrangement. Among patients with non‐squamous patients, univariate analyses showed that the *ALK* rearrangement rate was significantly higher in patients who were ≤60 years old (*P* < 0.001), female (*P* = 0.007), and without a smoking history (*P* = 0.001) (Table 7). In multivariate analysis, only younger age (HR = 2.7, 95%CI = 1.7–4.2, *P* < 0.001) was an independent predictor of *ALK* rearrangement. Among patients with squamous, four cases involved IHC‐confirmed ALK, with negative FISH results in two cases and no FISH testing in the other two cases. All four patients were ≤60 years old, three were male, and three had smoking history.

**Table 6:** Fisher exact probability method analysis of ALK rearrangement and clinical characteristics in 1134 NSCLC patients

Characteristics	No.	Wild type	Mutant type	*P*‐value
Age (N, %)				
≤60 years old	548 (48.0%)	473 (86.2%)	75 (13.8%)	<0.001
>60 years old	586 (52.0%)	554 (94.6%)	32 (5.4%)	
Gender (N,%)				
Male	642 (56.6%)	597 (93.0%)	45 (7.0%)	0.002
Female	492 (43.4%)	430 (87.4%)	62 (12.6%)	
Smoking history (N, %)				
No	588 (51.9%)	513 (87.2%)	75 (12.8%)	<0.001
Yes	515 (45.4%)	484 (94.0%)	31 (6.0%)	
Unknown	31 (2.7%)	30 (96.8%)	1 (3.2%)	
Pathology (N, %)				
Non‐squamous	1025 (90.4%)	922 (90.0%)	103 (10.0%)	0.025
Squamous	109 (9.6%)	105 (96.3%)	4 (3.7%)	
T stage (N, %)				
1	153 (13.5%)	135 (88.2%)	18 (11.8%)	0.586
2	268 (23.6%)	243 (90.7%)	25 (9.3%)	
3	107 (9.4%)	94 (87.9%)	13 (12.1%)	
4	526 (46.4%)	482 (91.6%)	44 (8.4%)	
x	80 (7.1%)	73 (91.3%)	7 (8.7%)	
N stage (N, %)				
0	161 (14.2%)	150 (93.2%)	11 (6.8%)	0.818
1	48 (4.2%)	44 (91.7%)	4 (8.3%)	
2	298 (26.3%)	269 (90.3%)	29 (9.7%)	
3	593 (52.3%)	533 (89.9%)	60 (10.1%)	
x	34 (3.0%)	31 (91.2%)	3 (8.8%)	
M stage (N,%)				
0	166 (14.6%)	150 (90.4%)	16 (9.6%)	0.886
1	968 (85.4%)	877 (90.6%)	91 (9.4%)	
Lung metastases				
No	724 (63.8%)	652 (90.1%)	72 (9.9%)	0.461
Yes	410 (36.2%)	375 (91.5%)	35 (8.5%)	
Bone metastases				
No	733 (64.6%)	656 (89.5%)	77 (10.5%)	0.111
Yes	401 (35.4%)	371 (92.5%)	30 (7.5%)	
Brain metastases				
No	948 (16.4%)	857 (90.4%)	91 (9.6%)	0.784
Yes	186 (83.6%)	170 (91.4%)	16 (8.6%)	
Adrenal metastases				
No	1046 (92.2%)	944 (90.2%)	102 (9.8%)	0.257
Yes	88 (7.8%)	83 (94.3%)	5 (5.7%)	
Liver metastases				
No	1040 (91.7%)	947 (91.1%)	93 (8.9%)	0.066
Yes	94 (8.3%)	80 (85.1%)	14 (14.9%)	
Pleural effusion				
No	813 (71.7%)	733 (90.2%)	80 (9.8%)	0.500
Yes	321 (28.3%)	294 (91.6%)	27 (8.4%)	
Pleural nodules				
No	1002 (88.4%)	910 (90.8%)	92 (9.2%)	0.428
Yes	132 (11.6%)	117 (88.6%)	15 (11.4%)	

ALK, anaplastic lymphoma kinase.

**Table 7:** Fisher exact probability method analysis of ALK rearrangement and clinical characteristics in 1025 non‐squamous NSCLC patients

Characteristics	No.	Wild type	Mutant type	*P*‐value
Age (N,%)				
<60 years old	509 (49.7%)	436 (85.7%)	73 (14.3%)	<0.001
≥60 years old	516 (50.3%)	486 (94.2%)	30 (5.8%)	
Gender (N,%)				
Male	550 (53.7%)	508 (92.4%)	42 (7.6%)	0.007
Female	475 (46.3%)	414 (87.2%)	61 (12.8%))	
Smoking history (N,%)				
No	565 (55.1%)	491 (86.9%)	74 (13.1%)	0.001
Yes	430 (42.0%)	402 (93.5%)	28 (6.5%)	
Unknown	30 (2.9%)	29 (96.7%)	1 (3.3%)	
T stage (N,%)				
1	148 (14.4%)	130 (87.8%)	18 (12.2%)	0.700
2	247 (24.1%)	222 (89.9%)	25 (10.1%)	
3	96 (9.4%)	84 (87.5%)	12 (12.5%)	
4	457 (44.6%)	416 (91.0%)	41 (9.0%)	
x	77 (7.5%)	70 (90.0%)	7 (9.1%)	
N stage (N,%)				
0	154 (15.0%)	143 (92.9%)	11 (7.1%)	0.736
1	42 (4.1%)	38 (90.5%)	4 (9.5%)	
2	263 (25.7%)	237 (90.1%)	26 (9.9%)	
3	533 (52.0%)	474 (88.9%)	59 (11.1%)	
x	33 (3.2%)	30 (90.9%)	3 (9.1%)	
M stage (N,%)				
0	121(11.8%)	108 (89.3%)	13 (10.7%)	0.749
1	904(88.2%)	814 (90.0%)	90 (10.0%)	
Lung metastases				
No	645 (62.9%)	576 (89.3%)	69 (10.7%)	0.391
Yes	380 (37.1%)	346 (91.1%)	34 (8.9%)	
Bone metastases				
No	650 (63.4%)	577 (88.8%)	73 (11.2%)	0.106
Yes	375 (36.6%)	345 (92.0%)	30 (8.0%)	
Brain metastases				
No	841 (82.0%)	754 (89.7%)	87 (10.3%)	0.589
Yes	184 (18.0%)	168 (91.3%)	16 (8.7%)	
Adrenal metastases				
No	942 (91.9%)	844 (89.6%)	98 (10.4%)	0.254
Yes	83 (8.1%)	78 (94.0%)	5 (6.0%)	
Liver metastases				
No	938 (91.5%)	849 (90.5%)	89 (9.5%)	0.061
Yes	87 (8.5%)	73 (83.9%)	14 (16.1%)	
Pleural effusion				
No	721 (70.3%)	645 (89.5%)	76 (10.5%)	0.495
Yes	304 (29.7%)	277 (91.1%)	27 (8.9%)	
Pleural nodules				
No	901 (87.9%)	813 (90.2%)	88 (9.8%)	0.425
Yes	124 (12.1%)	109 (87.9%)	15 (12.1%)	

ALK, anaplastic lymphoma kinase.

### First‐line targeted therapy rate

First‐line targeted therapy was 73.8% (340/461) for patients harboring *EGFR* mutations and 51.4% (55/107) for patients with *ALK* rearrangements. There was a negative correlation between the first‐line targeted therapy rate and the *EGFR* mutation detection period (r = −0.152, *P* = 0.02), while no significant correlation was detected among patients with ALK rearrangement (r = −0.179, *P* = 0.076).

## Discussion

The present study revealed that although adenocarcinoma was the most common pathological type to be submitted for *EGFR/ALK* evaluation, patients with squamous carcinoma had an *EGFR* mutation rate of 8.3% and an *ALK* rearrangement rate of 3.7%. Because of the relatively high mutation rate, patients with squamous cell carcinoma should also be routinely tested to determine their *EGFR* and *ALK* statuses.

Among four patients with squamous harboring *ALK* rearrangement, two showed inconsistent test results (positive for IHC and negative for FISH). Thus, IHC and FISH testing appear to provide inconsistent results regarding the squamous NSCLC patient's *ALK* status. Nevertheless, further validation of this result is needed, given the small sample size of this subgroup in the present study. A previous report regarding *ALK* rearrangement in NSCLC indicated that FISH provides higher sensitivity and specificity than IHC regarding the effects of targeted therapy, and that the interpretation of FISH results is more objective than that of IHC results.^17^ However, that study included a much smaller number of squamous cell carcinoma cases than adenocarcinoma cases (303 vs. 25 596). Therefore, further studies are needed to examine whether IHC and/or FISH are the most appropriate techniques for determining the *ALK* status, especially for patients with squamous cell carcinoma.

The present study revealed that the overall *EGFR* mutation rate was 40.7%, and gender, smoking history, and histology were independent predictors of *EGFR* mutation. These findings are consistent with the results of previous studies.^13^, ^14^, ^18^, ^19^, ^20^ Furthermore, we found that patients with *EGFR* mutations were more likely to have baseline brain metastases, which may be related to the downstream effects of *EGFR* on brain metastases. It is reported that *EGFR* inhibition decreased the rate of brain metastases in human DMA‐MB‐231 breast cancer cell lines. Although the patients with NSCLC were not evaluated, results suggested that *EGFR* may affect the phosphoinositide 3 kinase/protein kinase B/phospholipase Cγ pathway and subsequently lead to brain metastasis.^21^ Another study revealed that *EGFR*‐TKI therapy induced *MET* expression and phosphorylation, which may be associated with subsequent brain metastases in patients with NSCLC.^22^ While this relationship can only explain the increase in brain metastases after *EGFR*‐TKI treatment, it does not explain the relationship between *EGFR* mutations and baseline brain metastasis. Therefore, further studies are needed to better understand the relationship between *EGFR* mutations and baseline brain metastasis, and clinicians should be aware of this relationship when they encounter cases of *EGFR*‐mutated NSCLC, or cases with brain metastasis.

This study observed the *ALK*‐rearrangement rate was 9.4%, which is much higher than that reported among Asian patients with NSCLC of 4.1–5%.^23^ This difference may be related to the clinicopathological characteristics of the included patients. Previous reports indicated an *ALK* rearrangement rate of 13.5% (19/141) among patients with NSCLC who were female, Asian, did not smoke or smoked relatively small amounts, and adenocarcinoma.^16^, ^24^, ^25^, ^26^ In the present study, 85.8% (973/1134) of the enrolled patients had adenocarcinoma, which may explain the relatively high *ALK* rearrangement rate. The present study also revealed that only age was independently associated with *ALK* rearrangement, which is in line with previous reports.^27^, ^28^

The study suggested that first‐line targeted therapy rate for patients with NSCLC with *EGFR*‐activating mutation or *ALK* rearrangement were still low. This relatively low *EGFR*‐TKI treatment rate could be explained by a long interval until the *EGFR* mutation is detected; thus, it may be possible to increase this rate by shortening the *EGFR* mutation detection period, which may be achieved by obtaining sufficient pathological specimens in a timely manner and popularizing *EGFR* gene detection technology. However, there was no significant correlation between the ALK rearrangement detection period and first‐line targeted therapy rate. Although the *ALK* rearrangement detection period was shorter than the *EGFR* mutation detection period (5 days vs. 7 days), the first‐line ALK‐TKIs treatment rate was lower than the first‐line EGFR‐TKIs treatment rate (51.4% vs. 73.8%). This finding may be related to limited access to ALK inhibitors, based on their high cost.

In addition to the important discoveries revealed in the present study, there are also some limitations. First, these data are preliminary, and thus additional follow‐up is needed to examine the effects of targeted therapy in cases of NSCLC harboring *EGFR* mutation or *ALK* rearrangement. Second, different centers had varying numbers of patients who were eligible for enrolment, and we were unable to perform regional subanalyses of *EGFR/ALK* status.

In summary, the present study demonstrated that patients with squamous cell carcinoma should also be routinely tested to determine their EGFR and ALK gene statuses. The first‐line targeted therapy rate remains low for Chinese patients with NSCLC harboring *EGFR*‐mutation and/or *ALK*‐rearrangement. First‐line targeted therapy for *EGFR*‐positive patients was negative associated with the time from the pathological diagnosis to EGFR gene status confirmation. Further research is needed to identify whether IHC and/or FISH are the most appropriate techniques for determining the *ALK* status of patients with squamous cell carcinoma.

## Disclosure

This study was funded by Betta Pharmaceuticals Co., Ltd. All authors declare that there are no conflicts of interest.
